# Perioperative flurbiprofen axetil use and postoperative intestinal complications in elderly patients undergoing radical colorectal surgery

**DOI:** 10.3389/fphar.2026.1818438

**Published:** 2026-09-14

**Authors:** Xue Tian, Weisi Ding, Kai Shen, Huixin Liu, Haiyan An, Yi Feng

**Affiliations:** 1 Department of Anesthesiology, Peking University People’s Hospital, Beijing, China; 2 Department of Gastroenterology Surgery, Peking University People’s Hospital, Beijing, China; 3 Department of Clinical Epidemiology and Biostatistics, Peking University People’s Hospital, Beijing, China

**Keywords:** anastomotic leak, colorectal cancer, flurbiprofen axetil, intestinal complications, inverse probability of treatment weighting, old age

## Abstract

**Objective:**

To investigate the association between perioperative flurbiprofen axetil administration and postoperative intestinal complications in elderly patients undergoing radical colorectal surgery.

**Methods:**

We retrospectively reviewed the medical records of elderly patients who underwent primary radical colorectal surgery at Peking University People’s Hospital between November 2018 and March 2022. Data on patient demographics, surgical characteristics, perioperative medication use, and postoperative intestinal complications were collected. Univariate and multivariate logistic regression analyses were conducted to identify factors associated with postoperative intestinal complications. To minimize potential confounding, inverse probability of treatment weighting (IPTW) based on propensity scores was applied.

**Results:**

A total of 453 patients were included in the final analysis, among whom 54 (11.9%) developed postoperative intestinal complications. These complications comprised postoperative ileus (36 pts, 7.9%), anastomotic leakage (17 pts, 3.8%), and chylous fistula (3 pts, 0.7%). Perioperative flurbiprofen axetil use was significantly associated with a higher risk of postoperative intestinal complications (*P* = 0.002; odds ratio [OR] = 3.005, 95% confidence interval [CI]: 1.487–6.072). After adjustment for baseline demographic characteristics and comorbidities using IPTW, flurbiprofen axetil administration remained independently associated with an approximately threefold increased risk of intestinal complications (IPTW-adjusted *P* = 0.005; odds ratio = 3.263, 95% confidence interval: 1.429–7.452).

**Conclusion:**

Perioperative use of flurbiprofen axetil may be associated with an increased risk of postoperative intestinal complications in elderly patients undergoing radical colorectal surgery. Further prospective studies with larger sample sizes and dose-stratified analyses are warranted to confirm these findings and clarify the underlying mechanisms.

## Highlights

What is known:

1. Radical colorectal surgery is an important treatment for colorectal diseases. Elderly patients are at higher risk of postoperative intestinal complications due to physiological decline and multiple comorbidities, and these complications are one of the main factors leading to poor postoperative prognosis in this population.
2. Flurbiprofen axetil is a commonly used non-steroidal anti-inflammatory analgesic in clinical practice and is widely applied for perioperative analgesia in surgery. However, the association between its perioperative use and postoperative intestinal complications in elderly patients undergoing radical colorectal surgery remains unclear without definite targeted research conclusions.

What is new:

1. Perioperative flurbiprofen axetil use is associated with an increased risk of postoperative intestinal complications in elderly patients undergoing radical colorectal surgery.
2. In addition to flurbiprofen axetil use, male sex and intraoperative fluid administration volume are independently associated with postoperative intestinal complications.
3. Further studies focusing on dosage and administration frequency may help clarify the relationship between flurbiprofen axetil and severe AL in elderly patients.

## Background

The clinical management of elderly patients has become an important priority in modern healthcare (Yun et al., 2025). Intestinal surgery can substantially affect quality of life and long-term survival, with particularly pronounced consequences in elderly populations (Johnson et al., 1995). Postoperative intestinal complications after radical colorectal surgery are associated with reduced 5-year overall survival and 3-year disease-free survival, prolonged hospital length of stay, and impaired postoperative quality of life (Arnarson et al., 2019). Among these complications, anastomotic leakage (AL) is considered the most severe and is strongly associated with prolonged hospitalization and increased mortality (Hirano et al., 2023). Nonsteroidal anti-inflammatory drugs (NSAIDs) are widely recommended for perioperative pain management, including in colorectal surgery (Gustafsson et al., 2019; Kianian et al., 2024). However, increasing evidence suggests that NSAIDs use may be associated with higher risks of postoperative complications, particularly AL (Gorissen et al., 2012) and acute kidney injury (AKI) (Wa et al., 2022). NSAIDs are commonly classified as non-selective agents inhibiting cyclooxygenase (COX)-1 and COX-2, or selective COX-2 inhibitors. Previous studies have reported an increased incidence of AL with diclofenac (Klein et al., 2012; Modasi et al., 2018) and celecoxib (Holte et al., 2009), whereas other reports have found no association between NSAID use and AL following anterior resection for rectal cancer (Rutegård et al., 2016; Kverneng Hultberg et al., 2017), highlighting ongoing controversy.

Flurbiprofen axetil is a non-selective NSAID with established analgesic and anti-inflammatory effects and is widely used for perioperative analgesia, particularly in patients unable to tolerate oral medications (Brogden et al., 1979; Wick et al., 2017). It is frequently incorporated into multimodal analgesia regimens to reduce opioid consumption and opioid-related adverse effects (Long et al., 2022; Liu et al., 2022). In our previous work, we observed a potential association between perioperative flurbiprofen axetil use and postoperative intestinal complications after colorectal surgery (Ding et al., 2025). As a result, flurbiprofen axetil is often used more cautiously in elderly patients in clinical practice. However, whether such caution is necessary and, if so, whether it is sufficient in elderly patients undergoing radical colorectal surgery remains unclear. Although a higher incidence of postoperative nausea has been reported in patients receiving flurbiprofen axetil (Song et al., 2022), evidence regarding its association with postoperative intestinal complications in elderly populations remains limited.

Therefore, evaluating the relationship between perioperative flurbiprofen axetil use and postoperative intestinal complications in elderly patients undergoing radical colorectal surgery is clinically important and may help inform safer and more individualized perioperative analgesic strategies in this vulnerable population.

## Methods

### Ethical considerations

This single-center retrospective cohort study was conducted at Peking University People’s Hospital (Beijing, China) on data retrieved from patients who underwent their first radical colorectal surgery between November 2018 and March 2022. The study was approved by the Medical Ethics Committee of Peking University People’s Hospital (Beijing, China) in 2024 (Approval No.: 2024PHB358-001; Chairperson: Dr. Xueguang Zhu), and all ethical standards were adhered to throughout the study. All procedures and protocols of this study were conducted in strict compliance with the ethical principles and moral norms outlined in the World Medical Association Declaration of Helsinki, as well as relevant national and institutional ethical guidelines.

### Inclusion and exclusion criteria

This study included patients aged ≥65 years who underwent elective radical colorectal resection. Inclusion criteria were as follows: elective (non-emergent) surgery, American Society of Anesthesiologists (ASA) physical status classification (Keats, 1978)I–III, and performance of radical colorectal resection.

Exclusion criteria were as follows: undergoing a subsequent procedure as part of a planned staged colorectal surgical strategy, such as stoma reversal, preoperative admission to the intensive care unit (ICU), missing or incomplete prognostic data, and receipt of neuraxial analgesia (as flurbiprofen axetil is typically avoided in these patients because of its potential effects on coagulation). In addition, patients with severe hepatic, renal, or coagulation dysfunction—conditions in which flurbiprofen axetil is contraindicated—were excluded (Bindu et al., 2020).

### Cohort overlap disclosure

Our team previously conducted a retrospective colorectal surgery study covering January 2018 to March 2022, which explored the association between flurbiprofen axetil and postoperative intestinal complications in all adult patients (Ding et al., 2025). Based on those preliminary results, we performed a focused analysis of elderly patients in the present study. To improve population homogeneity, we narrowed the study period to November 2018–March 2022. While the two studies have overlapping timeframes, their cohorts are entirely distinct.

### Patient characteristics

Baseline patient characteristics were collected, including age, gender, body mass index (BMI), ASA classification, and comorbidities (hypertension, diabetes, coronary heart disease, and pulmonary disease, *etc.*). Medication history was recorded, including use of antiplatelet or anticoagulant agents, preoperative chemotherapy, antihypertensive medications, and steroid hormones.

Preoperative laboratory data were extracted from the most recent laboratory tests prior to surgery and included serum albumin, sodium, and hemoglobin (Hb) concentration. Preoperative anemia was defined as Hb < 13 g/dL in males and Hb < 12 g/dL in females (Pasricha et al., 2024).

In addition to baseline characteristics, detailed perioperative variables were collected, including surgical duration, use of laparoscopy, construction of a prophylactic stoma, application of regional block techniques, intraoperative fluid administration volume, estimated blood loss, vasopressor use, perioperative flurbiprofen axetil administration, intraoperative opioid consumption, maximum tumor diameter, lymph node metastasis, presence of intravascular tumor thrombus, and postoperative disposition (general ward or ICU).

### Anesthetic procedure

All patients received standardized general anesthesia. Anesthesia was induced with intravenous propofol (1.5–2.5 mg/kg), sufentanil (0.3 μg/kg), and rocuronium (0.6 mg/kg). Maintenance of anesthesia was achieved with sevoflurane or desflurane combined with continuous infusion of propofol and remifentanil, with the bispectral index (BIS) maintained between 40 and 60. Flurbiprofen axetil was the only NSAID administered perioperatively, and its use was jointly determined by anesthesiologists and surgeons based on individual patient comorbidities and surgical considerations. After surgery, patients were either extubated in the operating room or post-anesthesia care unit (PACU) or transferred to the ICU with the endotracheal tube *in situ*. Extubation was performed only after recovery of consciousness, stable respiration and circulatory function, and adequate neuromuscular strength, after which patients were transferred to the general ward. Postoperative analgesia was provided via patient-controlled analgesia (PCA), with opioids as the sole analgesic agents.

Vasopressor agents were administered at the discretion of the attending anesthesiologist based on baseline and intraoperative blood pressure measurements. No significant differences were observed between the flurbiprofen axetil (FBP) and non-flurbiprofen axetil (N-FBP) groups regarding the distribution of colorectal surgical procedures (*P* = 0.269) or anastomotic techniques. The choice of anastomotic method (end-to-end or end-to-side) was determined by the operating surgeon according to tumor location. Importantly, all procedures were performed by the same surgical team, minimizing potential operator-related confounding.

### Outcomes

The primary outcome was postoperative intestinal complications, including anastomotic leakage (AL), ileus, and chylous fistula. AL was defined according to the modified criteria proposed by Reisinger et al. (Reisinger et al., 2014), named the extraluminal presence of contrast medium or leakage of intestinal contents, accompanied by evidence of extravasation, intra-abdominal collections, or free gas confirmed by contrast-enhanced computed tomography (CT), contrast enema, re-laparotomy, or endoscopy, and requiring reintervention or targeted treatment. In-hospital anastomotic leakage postoperatively was included. AL was further classified using a validated grading system (Rahbari et al., 2010): Grade A (no active therapeutic intervention required), Grade B (active therapeutic intervention required), and Grade C (reoperation required). Grades B and C were defined as severe AL.

Prolonged postoperative ileus was defined as a pathological delay in the recovery of bowel function that met all the following standardized clinical criteria: inability to resume oral feeding by postoperative day 4; persistent abdominal distension, nausea or vomiting accompanied by an absence of spontaneous defecation and flatus; clinical necessity for nasogastric decompression or prolonged bowel rest; and radiographic confirmation to rule out mechanical intestinal obstruction. (Vilz et al., 2017). Postoperative chylous fistula was defined as the presence of persistent milky/creamy abdominal drainage after colorectal surgery, with drainage fluid triglyceride concentration ≥110 mg/dL (Besselink et al., 2016). Patients with infectious effusion, purulent exudate, hemorrhagic fluid, or transient physiological abdominal drainage were excluded (Baek et al., 2013).

All postoperative complications were diagnosed by attending gastrointestinal surgeons based on clinical presentation and imaging studies, including CT.

### Statistical analyses

Statistical analyses were performed using IBM SPSS Statistics version 26.0 (IBM Corp., Armonk, NY, USA). Continuous variables were presented as medians with interquartile ranges (IQRs) and compared using nonparametric tests; the Mann-Whitney U test was applied for two-group comparisons. Categorical variables were expressed as numbers and percentages and compared using the chi-square test or Fisher’s exact test, as appropriate. Given the large number of potential covariates, univariate analyses were first conducted to identify candidate risk factors for postoperative intestinal complications. Variables with a P value <0.1 in univariate analyses were entered into multivariate logistic regression models to avoid exclusion of potential confounders. A two-sided P value <0.05 was considered statistically significant. Results were reported as odds ratios (ORs) with 95% confidence intervals (CIs). To further adjust for baseline imbalances and potential confounding, inverse probability of treatment weighting (IPTW) based on propensity scores was applied.

## Results

### Demographic and baseline characteristics

A total of 491 elderly patients were initially screened for eligibility. After exclusion of 38 patients-7 due to missing clinical data, 2 due to preoperative ICU admission, 8 who received neuraxial anesthesia, and 21 with severe hepatic or renal dysfunction, coagulation disorders, or hypersensitivity to NSAIDs-453 patients were included in the final analysis (Figure 1). The study cohort comprised 268 men (59.2%) and 185 women (40.8%). Patients were stratified into two groups according to perioperative flurbiprofen axetil use: the flurbiprofen axetil group (FBP, n = 61, 13.5%) and the non-flurbiprofen axetil group (N-FBP, n = 392, 86.5%). Among patients in the FBP group, flurbiprofen axetil was administered intraoperatively in 41 patients (9.1% of the total cohort), postoperatively in 23 patients (5.1%), and both intraoperatively and postoperatively in 3 patients (0.7%). 10 patients (43.5%) received only a single 50 mg dose on postoperative day (POD) 1; 19 patients (82.6%) initiated postoperative medication within POD 3; only 4 patients (17.4%) started administration after postoperative day 7. Before weighting, comparison of baseline characteristics between the FBP and N-FBP groups showed that 15 variables had standardized mean difference (SMD) between 0.1 and 0.2, and 3 variables had SMD ≥0.2. After IPTW adjustment, balance between groups improved substantially: five variables had residual SMDs between 0.1 and 0.2 (ASA status classification, tumor vascular emboli, hypertension, coronary heart disease, and preoperative steroid use), while the SMD for preoperative angiotensin-converting enzyme inhibitor/angiotensin II receptor blockers (ACEI/ARB) use remained at 0.2 (Table 1).

**FIGURE 1:**
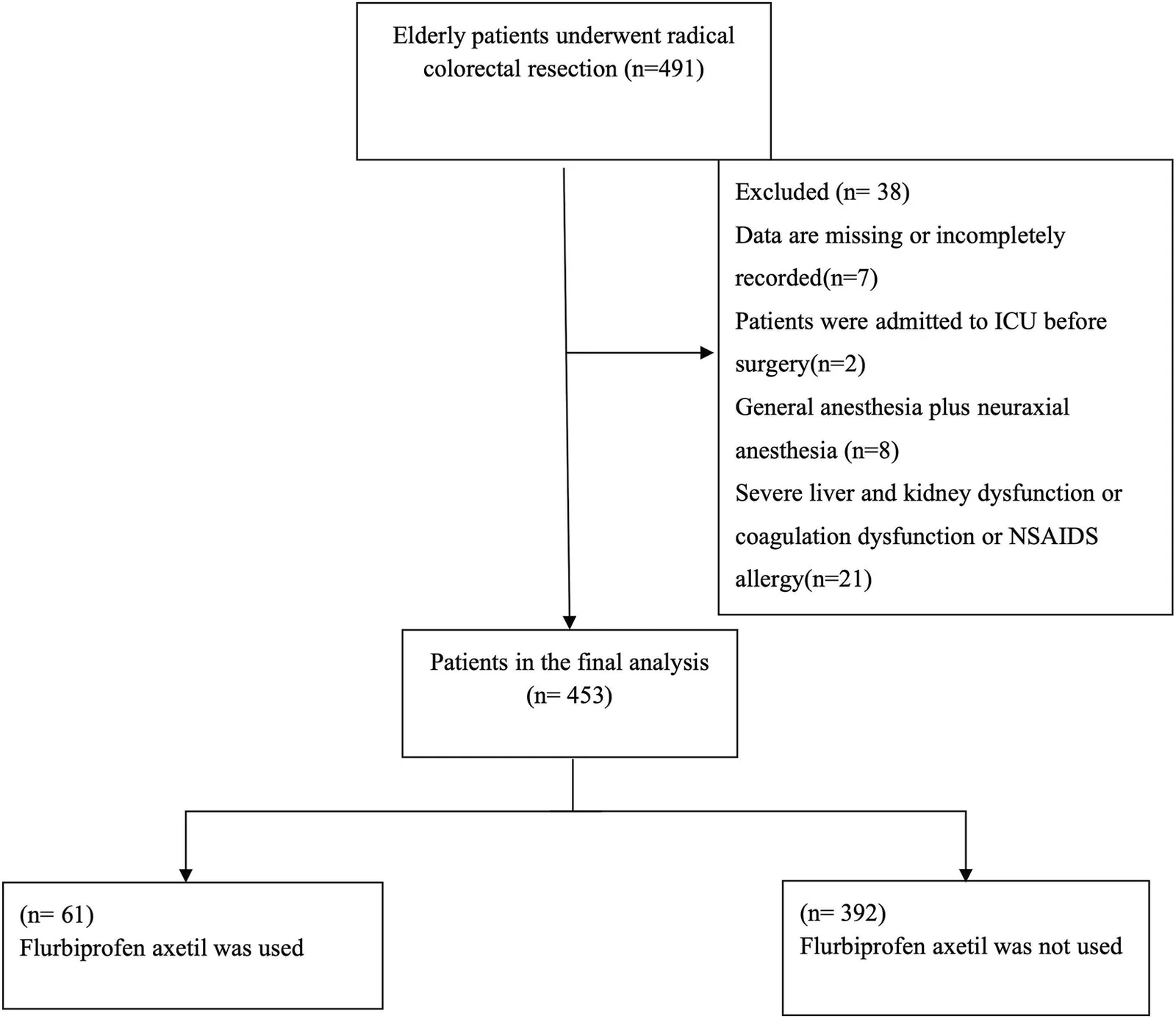
Process of data filtering used in the present study.

**TABLE 1:** Comparison of patient demographics, intraoperative findings, and preoperative history before and after IPTW between groups.

Variables	IPTW					
	FBP (n = 61)	N-FBP (n = 392)	*P* value	SMD	*P* value	SMD
*Patient characteristics*						
Age, year^*^	73 (11)	72 (10)	0.616	0.070	0.968	0.006
Female, n (%)	21 (34.4)	164 (41.8)	0.273	0.153	0.621	0.077
BMI, n (%)	​	​	0.425	0.113	0.688	0.058
* ≥ 28 kg/m* ^*2*^	9 (14.8)	44 (11.2)	​	​	​	​
* <28 kg/m* ^*2*^	52 (85.2)	348 (88.8)	​	​	​	​
ASA classification, n (%)	​	​	0.281	0.148	0.367	0.138
*Grade III*	30 (49.2)	164 (41.8)	​	​	​	​
* < Grade III*	31 (50.8)	228 (58.2)	​	​	​	​
*Surgery and anesthesia*, n (%)						
Duration	​	​	0.859	0.024	0.584	0.082
*> 3h*	39 (63.9)	246 (62.8)	​	​	​	​
*≤ 3h*	22 (36.1)	146 (37.2)	​	​	​	​
Surgical method	​	​	0.307	0.138	0.946	0.010
*Laparoscopy*	39 (63.9)	276 (70.4)	​	​	​	​
*Open abdomen*	22 (36.1)	116 (29.6)	​	​	​	​
Vasopressor	24 (39.3)	182 (46.4)	0.301	0.144	0.801	0.039
Intraoperative fluid infusion volume (L)^*^	2.4 (0.8)	2.1 (1.0)	0.204	0.043	0.794	0.031
Intraoperative blood loss	​	​	0.541	0.084	0.921	0.015
*<100 mL*	32 (52.5)	222 (56.6)	​	​	​	​
*≥100 mL*	29 (47.5)	170 (43.4)	​	​	​	​
Use regional block	28 (45.9)	154 (39.3)	0.327	0.134	0.780	0.042
Postoperative return	​	​	0.178	0.184	0.768	0.044
*The ward*	31 (50.8)	235 (59.9)	​	​	​	​
*ICU*	30 (49.2)	157 (40.1)	​	​	​	​
Lymph node metastasis	18 (29.5)	141 (36.0)	0.325	0.138	0.777	0.045
Tumor emboli in blood vessels	12 (19.7)	85 (21.7)	0.722	0.050	0.478	0.106
Maximum diameter of tumor	​	​	0.116	0.214	0.711	0.055
*<5 cm*	33 (54.1)	253 (64.5)	​	​	​	​
*≥5 cm*	28 (45.9)	139 (35.5)	​	​	​	​
Intraoperative opioids consumption (mg)^*^	30 (21)	35 (18)	0.143	0.029	0.966	0.009
*Previous history*, n (%)						
Hypertension	33 (54.1)	186 (47.4)	0.334	0.133	0.451	0.116
Diabetes	17 (27.9)	96 (24.5)	0.570	0.077	0.795	0.038
Coronary heart disease	14 (23.0)	69 (17.6)	0.315	0.133	0.442	0.118
Chronic lung disease	6 (9.8)	15 (3.8)	0.038	0.240	0.608	0.068
Albumin^*^	38.6 (3.80)	38.4 (4.98)	0.529	0.136	0.509	0.095
Hb < 12 g/dL in female, Hb < 13 g/dL in male	24 (39.3)	193 (49.1)	0.150	0.200	0.949	0.010
Na^+^ ^*^	141.4 (3.13)	141.3 (3.47)	0.974	0.014	0.858	0.027
*Preoperative medication*, n (%)						
Antiplatelet or anticoagulant therapy	15 (24.6)	67 (17.1)	0.157	0.185	0.836	0.027
Chemotherapy	8 (13.1)	37 (9.4)	0.372	0.116	0.812	0.032
ACEIs/ARBs	11 (18.0)	48 (12.2)	0.212	0.162	0.198	0.200
Statins	12 (19.7)	50 (12.8)	0.144	0.189	0.826	0.030
Other antihypertensive drugs	22 (36.1)	133 (33.9)	0.743	0.045	0.826	0.034
Diuretics	2 (3.3)	7 (1.8)	0.776	0.095	0.860	0.019
Steroid hormones	5 (8.2)	42 (10.7)	0.549	0.086	0.343	0.136

^*^ M(IQR) IPTW, inverse probability of treatment weighting; SMD, standardized mean difference; FBP, flurbiprofen axetil; BMI, body mass index; ASA, american society of anesthesiologists; ICU, intensive care unit; ACEIs, Angiotensin-converting enzyme inhibitors; ARBs, Angiotensin-receptor blockers.

Continuous measurement data were expressed as median (interquartile range, IQR), compared by non-parametric test, specifically the Mann-Whitney U test in two groups. Categorical variables were expressed as percentages, and comparisons between groups were using the Chi-square test or Fisher’s exact probability method before and after IPTW.

### Postoperative intestinal complications

Overall, postoperative intestinal complications occurred in 54 patients (11.9%). Of the 54 patients who developed composite endpoint events, a total of 56 individual complications were observed. Specifically, two patients concurrently suffered from both anastomotic leakage and postoperative ileus. Among these patients, 7 (13.0%) had received intraoperative flurbiprofen axetil and 7 (13.0%) had received postoperative flurbiprofen axetil. In univariate analysis, perioperative flurbiprofen axetil use, operative duration, intraoperative vasopressor administration, and coronary heart disease were associated with postoperative intestinal complications. Multivariate logistic regression identified perioperative flurbiprofen axetil use and intraoperative vasopressor administration as independent risk factors. Specifically, perioperative flurbiprofen axetil use was associated with a significantly increased risk of postoperative intestinal complications (*P* = 0.002; odds ratio [OR] = 3.005, 95% confidence interval [CI]: 1.487–6.072), as was intraoperative vasopressor administration (*P* = 0.033; OR = 1.908, 95% CI: 1.055–3.450) (Table 2).

**TABLE 2:** Univariate and multivariate analysis of intestinal complications.

Variables	Intestinal complications (n = 54)	No complications (n = 399)	Univariate *P* value	*Multivariate analysis*			
				*CI*			
				*OR*	*Lower*	*Upper*	*P value*
*Patient characteristics*							
Age (year)^*^	71 (9)	73 (10)	0.294	​	​	​	​
Females, n (%)	19 (35.2)	166 (41.6)	0.368	​	​	​	​
BMI, n (%)	​	​	0.552	​	​	​	​
*≥ 28 kg/m* ^*2*^	5 (9.3)	48 (12.0)	​	​	​	​	​
*<28 kg/m* ^*2*^	49 (90.7)	351 (88.0)	​	​	​	​	​
ASA, n (%)	​	​	0.227	​	​	​	​
*Grade III*	19 (35.2)	175 (43.9)	​	​	​	​	​
*< Grade III*	35 (64.8)	224 (56.1)	​	​	​	​	​
*Surgery and anesthesia*, n (%)							
Flurbiprofen axetil was used	14 (25.9)	47 (11.8)	0.004	3.005	1.487	6.072	0.002
Duration	​	​	0.070	1.765	0.919	3.391	0.088
*> 3h*	40 (74.1)	245 (61.4)	​	​	​	​	​
*≤ 3h*	14 (25.9)	154 (38.6)	​	​	​	​	​
Surgical method	​	​	0.863	​	​	​	​
*Laparoscopy*	37 (68.5)	278 (69.7)	​	​	​	​	​
*Open abdomen*	17 (31.5)	121 (30.3)	​	​	​	​	​
Use vasopressor during operation	32 (59.3)	174 (43.6)	0.030	1.908	1.055	3.450	0.033
Intraoperative fluid infusion volume (L)^*^	2.1 (1.0)	2.1 (1.0)	0.213	​	​	​	​
Intraoperative blood loss	​	​	0.338	​	​	​	​
*<100 mL*	27 (50.0)	227 (56.9)	​	​	​	​	​
*≥100 mL*	27 (50.0)	172 (43.1)	​	​	​	​	​
Use regional block during operation	20 (37.0)	162 (40.6)	0.616	​	​	​	​
Postoperative return	​	​	0.206	​	​	​	​
*The ward*	36 (66.7)	230 (57.6)	​	​	​	​	​
*ICU*	18 (33.3)	169 (42.4)	​	​	​	​	​
Lymph node metastasis	22 (40.7)	137 (34.3)	0.355	​	​	​	​
Tumor emboli in blood vessels	15 (27.8)	82 (20.6)	0.224	​	​	​	​
Maximum diameter of tumor	​	​	0.566	​	​	​	​
*<5 cm*	36 (66.7)	250 (62.7)	​	​	​	​	​
*≥5 cm*	18 (33.3)	149 (37.3)	​	​	​	​	​
Intraoperative opioids consumption (mg)^*^	35 (21)	35 (20)	0.170	​	​	​	​
*Previous history*, n (%)							
Hypertension	26 (48.1)	193 (48.4)	0.975	​	​	​	​
Diabetes	10 (18.5)	103 (25.8)	0.245	​	​	​	​
Coronary heart disease	5 (9.3)	78 (19.5)	0.067	0.416	0.157	1.105	0.078
Chronic lung disease	2 (3.7)	19 (4.8)	0.998	​	​	​	​
Albumin^*^	37.5 (5.0)	38.5 (4.8)	0.341	​	​	​	​
Hb < 12 g/dL in female, Hb < 13 g/dL in male	24 (44.4)	193 (48.4)	0.588	​	​	​	​
Na^+^ ^*^	141.1 (3.8)	141.4 (3.4)	0.246	​	​	​	​
*Preoperative medication*, n (%)							
Antiplatelet or anticoagulant therapy	10 (18.5)	72 (18.0)	0.932	​	​	​	​
Chemotherapy	5 (9.3)	40 (10.0)	0.860	​	​	​	​
ACEIs/ARBs	9 (16.7)	50 (12.5)	0.397	​	​	​	​
Statins	8 (14.8)	54 (13.5)	0.797	​	​	​	​
Other antihypertensive drugs	19 (35.2)	136 (34.1)	0.873	​	​	​	​
Diuretics	1 (1.9)	8 (2.0)	>0.999	​	​	​	​
Steroid hormones	8 (14.8)	39 (9.8)	0.254	​	​	​	​

^*^ M(IQR) BMI, body mass index; ASA, american society of anesthesiologists; ICU, intensive care unit; ACEIs, Angiotensin-converting enzyme inhibitors; ARBs, Angiotensin-receptor blockers.

Continuous measurement data were expressed as median (interquartile range, IQR), categorical variables were expressed as percentages; Univariate *P* value in univariate logistic regression analysis, multivariate analysis *P* value in multivariate logistic regression analysis with *P* < 0.1 in univariate logistic regression analysis.

After IPTW adjustment, multivariable logistic regression demonstrated that gender, perioperative flurbiprofen axetil use, and intraoperative fluid infusion volume were independently associated with postoperative intestinal complications. Male gender was associated with a higher risk (*P* = 0.004; OR = 3.865, 95% CI: 1.551–9.631), as was perioperative flurbiprofen axetil administration (*P* = 0.005; OR = 3.263, 95%CI: 1.429–7.452). Besides, intraoperative fluid infusion volume was associated with postoperative intestinal complications (*P* = 0.002; OR = 0.464, 95%CI: 0.286–0.753) (Table 3).

**TABLE 3:** Multivariate analysis of intestinal complications after IPTW.

Variables	​	CI		​	​	​	​
	OR	Lower	Upper	*P* value	Beta	SE	Wald
*Patient characteristics*							
Age	0.960	0.887	1.039	0.314	−0.041	0.040	1.014
Gender	3.865	1.551	9.631	0.004	1.352	0.465	8.469
BMI	0.292	0.056	1.526	0.144	−1.232	0.842	2.143
ASA	0.759	0.279	2.064	0.588	−0.276	0.509	0.294
*Surgery and anesthesia*							
Flurbiprofen axetil was used	3.263	1.429	7.452	0.005	1.183	0.420	7.928
Duration	1.820	0.662	5.001	0.245	0.599	0.514	1.356
Surgical method	1.022	0.396	2.637	0.963	0.022	0.482	0.002
Use vasopressor during operation	1.474	0.412	5.273	0.550	0.388	0.649	0.358
Intraoperative fluid infusion volume	0.464	0.286	0.753	0.002	−0.768	0.247	9.706
Intraoperative blood loss	1.806	0.768	4.249	0.175	0.591	0.435	1.844
Use regional block during operation	0.709	0.278	1.808	0.471	−0.344	0.476	0.521
Postoperative return	1.201	0.304	4.745	0.794	0.183	0.699	0.069
Lymph node metastasis	1.587	0.624	4.032	0.331	0.462	0.474	0.947
Tumor emboli in blood vessels	1.087	0.330	3.583	0.891	0.083	0.607	0.019
Maximum diameter of tumor	1.226	0.423	3.554	0.707	0.204	0.542	0.142
Intraoperative opioids consumption	1.007	0.993	1.022	0.326	0.007	0.007	0.967
*Previous history*							
Hypertension	0.786	0.244	2.535	0.686	−0.241	0.596	0.164
Diabetes	0.746	0.289	1.928	0.544	−0.293	0.483	0.368
Coronary heart disease	0.436	0.054	3.503	0.434	−0.830	1.060	0.613
Chronic lung disease	0.872	0.153	4.958	0.877	−0.137	0.884	0.024
Albumin	0.935	0.817	1.069	0.325	−0.068	0.069	0.972
Hb < 12 g/dL in female, Hb < 13 g/dL in male	0.636	0.188	2.144	0.464	−0.453	0.619	0.537
Na^+^	0.964	0.816	1.139	0.666	−0.037	0.085	0.187
*Preoperative medication*							
Antiplatelet or anticoagulant therapy	0.845	0.219	3.261	0.806	−0.169	0.687	0.060
Chemotherapy	2.896	0.820	10.232	0.099	1.063	0.642	2.741
ACEIs/ARBs	1.878	0.658	5.360	0.238	0.630	0.534	1.393
Statins	1.112	0.205	6.045	0.902	0.107	0.861	0.015
Other antihypertensive drugs	2.269	0.739	6.972	0.152	0.819	0.571	2.059
Diuretics	1.536	0.203	11.636	0.677	0.429	1.030	0.174
Steroid hormones	3.370	0.961	11.810	0.058	1.215	0.638	3.625

BMI, body mass index; ASA, american society of anesthesiologists; ICU, intensive care unit; ACEIs, Angiotensin-converting enzyme inhibitors; ARBs, Angiotensin-receptor blockers.

Multivariate logistic regression analysis after IPTW.

### AL, ileus and chylous fistula

Among the 54 patients with postoperative intestinal complications, 36 (7.9% of the total cohort) developed postoperative ileus, 17 (3.8%) developed anastomotic leakage (AL), and 3 (0.7%) developed chylous fistula (Table 4). The median length of hospital stay was no differ in the FBP group and in the N-FBP group (16 (7) days vs. 16 (6) days, *P* > 0.05). Separate chi-square analyses demonstrated a significant association between perioperative flurbiprofen axetil use and postoperative AL (*P* = 0.020). No significant associations were observed between flurbiprofen axetil use and postoperative ileus or chylous fistula.

**TABLE 4:** Postoperative intestinal-related complications.

Postoperative complications	n (%)	FBP, n (%)	N-FBP, n (%)	χ^2^ (df)	*P* value
Ileus	36 (7.9)	8 (13.1)	28 (7.1)	1.822 (1)	0.177
AL	17 (3.8)	6 (9.8)	11 (2.8)	5.381 (1)	0.020
chylous fistula	3 (0.66)	0 (0)	3 (0.77)	0.871 (1)	0.351

FBP, flurbiprofen axetil; AL, anastomotic leak.

Comparisons between groups were using the Chi-square test or Fisher’s exact probability method.

Postoperative AL occurred in 17 patients. Of these, 4 patients (23.5%) received intraoperative flurbiprofen axetil and 2 patients (11.8%) received postoperative flurbiprofen axetil. According to severity grading, 5 patients (29.4%) developed grade A AL, among whom 1 patient received intraoperative flurbiprofen axetil (100 mg). 4 patients (23.5%) developed grade B AL, 3 of whom received intraoperative flurbiprofen axetil (100 mg). 8 patients (47.1%) developed grade C AL requiring reoperation; 2 of these patients received perioperative flurbiprofen axetil, both administered postoperatively with more than one dose (Table 5).

**TABLE 5:** Proposal for the definition and severity grading of anastomotic leakage (Rahbari et al., 2010).

Definition	Defect of the intestinal wall integrity at the colorectal or colo-anal anastomotic site (including suture and staple lines of neorectal reservoirs) leading to a communication between the intra- and extraluminal compartments. A pelvic abscess close to the anastomosis is also considered as anastomotic leakage	
Grade	A	Anastomotic leakage requiring no active therapeutic intervention
	B	Anastomotic leakage requiring active therapeutic intervention but manageable without re-laparotomy
	C	Anastomotic leakage requiring re-laparotomy

Postoperative ileus was diagnosed in 36 patients, including 8 patients who received flurbiprofen axetil (4 intraoperatively and 4 postoperatively), with no statistically significant association observed (*P* = 0.177). Postoperative chylous fistula occurred in 3 patients, none of whom received flurbiprofen axetil perioperatively (*P* = 0.351).

## Discussion

This study examined postoperative intestinal complications—including anastomotic leakage (AL), ileus, and chylous fistula—in elderly patients undergoing radical colorectal surgery, with a particular focus on perioperative flurbiprofen axetil use. Postoperative intestinal complications, especially AL, are known to adversely affect postoperative recovery and long-term outcomes in elderly patients (Gorissen et al., 2012; Chen et al., 2025), underscoring the clinical importance of identifying potentially modifiable perioperative risk factors.

In this cohort, perioperative flurbiprofen axetil administration was significantly associated with an increased risk of postoperative intestinal complications. This association remained robust in analyses adjusted using inverse probability of treatment weighting (IPTW), suggesting that the observed relationship was not solely attributable to measured baseline imbalances. Given the widespread use of flurbiprofen axetil as part of multimodal analgesia in colorectal surgery, this finding warrants careful consideration.

To further characterize this association, flurbiprofen axetil exposure was stratified by timing (intraoperative vs. postoperative) and dosing frequency (single vs. multiple doses). Although postoperative administration generally involved higher cumulative doses than intraoperative use, no clear dose–response relationship was identified. This may reflect limited statistical power due to the relatively small number of exposed patients, rather than the absence of a true biological effect.

AL represents the most severe postoperative intestinal complication after colorectal surgery and is associated with increased morbidity and mortality (Penna et al., 2019). In the present study, perioperative flurbiprofen axetil use was associated with postoperative AL in unadjusted analyses. However, the low incidence of AL and the small number of events within severity subgroups precluded definitive conclusions regarding causality or severity-specific effects. Nonetheless, the observed exposure patterns suggest that future studies focusing on dosage and administration frequency may help clarify the relationship between flurbiprofen axetil and severe AL in elderly patients.

The potential link between flurbiprofen axetil and intestinal complications is biologically plausible. As a non-selective NSAID, flurbiprofen axetil inhibits both COX-1 and COX-2. While COX-2 inhibition mediates analgesic and anti-inflammatory effects, COX-1 inhibition may compromise intestinal mucosal protection by reducing prostaglandin synthesis, impairing microvascular blood flow, and increasing intestinal permeability (Chen et al., 2021; Kaur and Singh, 2022).

In addition, NSAIDs can induce mitochondrial dysfunction and epithelial apoptosis through non-COX–dependent mechanisms, further contributing to mucosal injury (Panchal and Prince Sabina, 2023). Flurbiprofen axetil’s lipid microsphere formulation facilitates accumulation at inflammatory sites, potentially enhancing both its analgesic efficacy and its impact on local prostaglandin activity (Yao et al., 2021; He Y. et al., 2023; Wang et al., 2021). Experimental data indicate that flurbiprofen markedly reduces mucosal prostaglandin levels, supporting the biological plausibility of the present clinical findings (Bjarnason et al., 2018; Mahmud et al., 1998).

In addition to flurbiprofen axetil use, male sex and intraoperative fluid administration volume were independently associated with postoperative intestinal complications. Male patients exhibited a higher risk, potentially reflecting sex-related anatomical or physiological differences, including variations in prostaglandin production (He J. et al., 2023; Frasson et al., 2015). Adequate intraoperative fluid administration is critical for maintaining tissue perfusion, particularly in elderly patients, and insufficient resuscitation may predispose to postoperative complications. Consistent with prior studies, strategies such as goal-directed hemodynamic therapy may reduce postoperative morbidity in high-risk surgical populations (Lim and Lee, 2019; Gurgel and do Nascimento, 2010; Hamilton et al., 2010).

Several limitations merit consideration. First, postoperative intestinal complications were captured only during hospitalization, potentially underestimating their true incidence. Second, flurbiprofen axetil was the only NSAID evaluated, and dosing was not standardized, precluding robust assessment of dose–response relationships. Third, emergency surgeries were not included, although NSAID use in urgent settings may further increase the risk of AL (Haddad et al., 2017).

Despite the application of IPTW to balance baseline confounding, mild residual covariate imbalance remained after weighting, with several variables and ACEI/ARB exposure showing SMD values up to 0.20. According to established propensity score methodological guidelines, SMD is recognized as a tolerable imbalance threshold for real-world observational studies, which does not completely undermine the validity of weighted comparative estimates (Lu et al., 2023). In this study, we adhered to a prespecified, clinically informed propensity score model without *post hoc* addition of nonlinear or interaction terms to avoid overfitting and arbitrary model tuning bias. Strict SMD balance (SMD <0.10) was not fully achieved, which may be attributed to the relatively limited single-center sample size. This residual confounding should be considered when interpreting the study results. Future investigations with larger sample sizes and multicenter prospective cohorts are warranted to optimize covariate balance and verify the robustness of the current findings.

In addition, confounding by indication cannot be completely eliminated, as flurbiprofen axetil administration was determined perioperatively by clinicians based on individual patient and surgical conditions. Although we performed rigorous covariate adjustment and IPTW analysis, unmeasured or incompletely quantified factors, including postoperative pain severity, hemodynamic fluctuation, renal function variation, ERAS protocol adherence, and temporal differences in surgical management across operative years, may still introduce residual confounding and bias the observed association and this single-center retrospective design limits generalizability.

In summary, perioperative flurbiprofen axetil use was associated with an increased risk of postoperative intestinal complications in elderly patients undergoing radical colorectal surgery. These findings highlight the need for careful risk–benefit assessment when selecting perioperative analgesic strategies in this vulnerable population and support the need for future prospective studies to clarify optimal NSAID use with respect to timing, dosage, and patient selection.

## Data Availability

The original contributions presented in the study are included in the article/supplementary material, further inquiries can be directed to the corresponding authors.

## Appendix

Supplemental Materials: Detailed IPTW Methodological Information.

This study employed conventional raw inverse probability of treatment weighting (IPTW), rather than stabilized IPTW, to estimate the population average treatment effect (ATE) of flurbiprofen axetil. Individual propensity scores were derived from a multivariable logistic regression model adjusted for comprehensive perioperative and baseline confounders, including age, sex, BMI, ASA classification, operation duration, surgical approach, intraoperative vasopressor use, fluid infusion, blood loss, nerve block application, lymph node dissection, thromboembolism prophylaxis, tumor size, postoperative opioid consumption, chronic comorbidities, serum biochemical indicators, preoperative chemotherapy, and routine perioperative medications.

The primary estimand of this analysis was the population-level ATE. Raw IPTW was calculated as 1/propensity score for patients receiving flurbiprofen axetil and 1/(1 − propensity score) for control patients, with propensity scores representing the individualized treatment probability conditional on all adjusted covariates. No weight truncation was performed, as the weight distribution ranged from 1.019 to 20.076 without extreme outliers, avoiding bias from extreme weight values. Robust sandwich standard errors were calculated via the survey package in R to correct weighting-related variance inflation and guarantee valid statistical inference for weighted analyses.
